# ‘Do you feel well or unwell?’ A study on children’s experience of estimating their nausea using the digital tool PicPecc

**DOI:** 10.1177/13674935221089746

**Published:** 2022-04-22

**Authors:** Linda Esplana, Malin Olsson, Stefan Nilsson

**Affiliations:** 1 Region Västra Götaland, 89599 Sahlgrenska University Hospital, Queen Silvia Children´s Hospital, Gothenburg, Sweden; 2 Institute of Health and Care Sciences, and University of Gothenburg Centre for Person-Centred Care (GPCC), 174416 Sahlgrenska Academy, University of Gothenburg, Gothenburg, Sweden

**Keywords:** child, interview, nausea, neoplasms, mobile applications, symptom assessment

## Abstract

Research with childhood cancer has progressed greatly in recent years, resulting in much improved treatment that is more intensive. However, with this new treatment children often experience negative symptoms, and research shows that nausea is a symptom that most affects them. Pictorial support in person-centred care for children (PicPecc) is a digital picture-based tool for children who undergo treatment due to their cancer diagnosis and helps them more effectively communicate and self-report their symptoms and emotions. The aim of the study was to investigate children’s experience of (i) using mHealth in nausea management and (ii) their acceptability of using an application (App). Semi-structured interviews were conducted with eight children aged five to fifteen years. Data were analysed with qualitative content analysis. The findings were presented in three categories: 1) Communicating feelings, 2) Playfulness generated in motivation and 3) App adaptable to children’s capabilities. Using an App contributed to new opportunities for the children to participate in their care. They experienced their treatment in different ways and used different strategies to manage and distract themselves from their symptoms. Using the PicPecc App can increase healthcare staff’s understanding of how children experience nausea when they undergo chemotherapy.

## Introduction

In Sweden, about 350 children under the age of 18 are diagnosed with cancer every year (Gustafsson et al., 2013). One third of these children suffer from leukaemia, one third from brain tumours and approximately 40% from some form of solid tumour (Barncancerregistret, 2020). Great progress has been made in childhood cancer research, and today about 85% of the children treated for cancer survive (Barncancerfonden, 2019). Chemotherapy, surgery and radiation therapy are the most common treatment methods and can be given in different combinations depending on the diagnosis and treatment protocol (Gabriel et al., 2019; Kattner et al., 2019). Aggressive chemotherapy and new research drugs have contributed to increased survival in children with cancer. As a result, the children often have problems with several severe side effects (Miller et al., 2011), such as mucositis and disturbance in food intake (Kamsvåg-Magnusson et al., 2014).

Decreased appetite, decreased energy, fatigue, hair loss, nausea, pain and weight loss have been documented as common side effects among children during cancer treatment (Baggott et al., 2009; Dupuis et al., 2016; Johnston et al., 2018). Of these symptoms, nausea has been reported as the symptom that affected the children’s well-being the most (Miller et al., 2011).

How symptom management of children with cancer can be optimized needs to be addressed (Dupuis et al., 2019).

Nausea assessment is an important strategy in nausea management (Evans et al., 2020). When research has compared how children rate themselves compared to how adults rate children, it turns out that adults rate children’s symptoms lower (Pinheiro et al., 2018). Linder et al. (2018) reported that children under the age of ten also had difficulty communicating what they felt or experienced verbally.

There are several types of rating scales designed according to one or more symptoms, and estimating pain is the most common among children (Bernier Carney et al., 2021; Zheng et al., 2020). Digital rating scales are constantly undergoing development (Cook et al., 2019; Hyslop et al., 2019) and clinical practice is in a paradigm shift toward digital tools for managing nausea (Wiljén et al., 2022). The digital era has given new opportunities to facilitate assessments in children, and using an application (App) has previously been reported to be effective and easy, as well as acceptable to children (Tiozzo et al., 2021).

Miller et al. (2011) state that if healthcare staff let the children estimate their nausea, they will have a greater opportunity to provide better and earlier symptom relief. Another study has shown that the symptom most affecting the child was the symptom the nurse became aware of via assessment (Hyslop et al., 2019). Woodgate and Degner (2003) found that children did not always tell their healthcare staff about their symptoms. Although mild or manageable symptoms did not always become known to the nurse, the children did receive symptom relief for more intense symptoms. The children also stated that they never got used to their symptoms but learned to adapt to them (Woodgate and Degner, 2003). It has been shown that better routines to draw attention to children’s moods are necessary in order to give them the help they need (Hyslop et al., 2019). A traditional rating scale could frustrate smaller children because it might not be adapted to the child’s development and ability and might therefore be difficult to understand (Thunberg et al., 2021). To avoid this, it is important that caregivers use creative assessment methods that are adapted to children (Linder et al., 2018). Knowledge regarding which nausea assessments are most effective is still sparse (Momani and Berry, 2017; Phillips et al., 2016).

This study would like to contribute by facilitating the care process and investigating how the child can be involved in his or her care whenever possible. A person-centred approach facilitates the care process and supports the child’s best interests (Coyne et al., 2018). Working in a person-centred way from the child’s perspective means giving the child the opportunity to talk about themselves and their wishes and providing the conditions for them to participate in their own care (Swedish institute for standards (SIS), 2020; Thunberg et al., 2021). By listening and focusing on the child’s story, a common understanding is created between the child and the healthcare staff. It is through the child being able to share their story that the partnership arises (Thunberg et al., 2021). By sharing experiences and knowledge, a common understanding is created of what the situation looks like and what resources are available (Thunberg et al., 2021). The child’s story and wishes should be documented in the medical record, as clear documentation helps the child and the healthcare staff to follow the plan and ensures continuity in care (SS-EN 17398:2020, 2020; Thunberg et al., 2021).

Pictorial support in person-centred care for children – PicPecc – is a digital App for mobile and tablet which helps children estimate their symptoms (Wiljén et al., 2022). The purpose is for children treated for cancer to be able to communicate their symptoms effectively with a digital, image-based communication support, providing them with greater opportunity for optimal symptom relief and increased well-being. The App can be used as an aid during procedures and various treatments and is intended to increase the child’s right to participate based on a person-centred approach (Wiljén et al., 2022).

In summary, nausea is a symptom that has large impact on children undergoing chemotherapy. Assessment of nausea is not frequently conducted in clinical practice, but with new technologies, nausea management can most probably be supported. There is nevertheless a gap in knowledge of how a digital platform can contribute to nausea management in children undergoing chemotherapy and therefore a need to describe the feasibility of an App with the potential to support such children.

### Aim

To investigate children’s experience of (i) using mHealth in nausea management and (ii) their acceptability of using an App.

## Methods

### Study design

This is a feasibility study in accordance with the UK Medical Research Council’s key principles for development and evaluation of complex interventions in healthcare (Skivington et al., 2021). A qualitative inductive interview study following the criteria for reporting qualitative research 32-item checklist.

### Recruitment

Purposeful sampling of children who had chemotherapy in their treatment schedule was conducted. The children were selected in consultation with the section leader at a Childhood Cancer Centre in West Sweden.

Inclusion criteria for the study were children between five and seventeen years of age undergoing chemotherapy for at least 3 days at the Childhood Cancer Centre. The children had to understand and make themselves understood in Swedish. Exclusion criteria were newly diagnosed patients.

### Data collection

Data collection took place via individual qualitative interviews by two interviewers specialized in paediatric nursing. It was important to include the children’s voices because the intervention was intended to shed light on *their* perspective on nausea (Nilsson et al., 2015). Article 12 of the Convention on the Rights of the Child emphasizes that healthcare staff must ensure that children form their own opinions and can express them freely whenever care affects them (United Nations, 1989). The interviews were based on an interview guide and conducted online using the videoconferencing system Zoom (Lobe et al., 2020). To ensure that the questions in the interview guide were relevant and communicated in a way that the children would understand, two pilot interviews were conducted. Two children aged eight and eleven were interviewed. These children did not have experience of childhood cancer themselves but were used as subjects to ensure that the language in the interview was adapted from a child’s perspective. The children were given access to the PicPecc App and had a say in its design. After the pilot interviews were conducted, the language was slightly adjusted to better suit the children in both the App and the interview guide. These pilot interviews were not included in the results.

This was the first time the children used the PicPecc App. All interviews apart from one were conducted within a week after chemotherapy treatment. Creative methods as aids are becoming increasingly common in research with children. These aids act as ‘triggers’, and can involve anything from painting or drawing around a subject, playing theatre, taking pictures or various objects that evoke memories and help children tell their story (Carter and Ford, 2013). To increase the child’s involvement in the study and help them remember the App and the various questions, the authors used image support during the interviews. Pictures from the App were printed out and served as an aid for the children to convey their stories during the interview.

The interviews were conducted with a parent helping the children connect to the technology if necessary. The children were able to choose for themselves whether a parent would be present for the interview or not. Only one of the first and the second author (L.E., M.O.) was present for each interview. All interviews were recorded, transcribed verbatim and de-identified. Each interview lasted between 15 and 30 min, with the average time being 20 min.

### Context

The context was the use of the PicPecc App during chemotherapy, with the aim of facilitating nausea management. The App was divided into image questions, text questions and a faces thermometer scale, with fields where the children could fill in their answers. Application managers can choose the design of their estimation template (Supplementary file 1, 2).

Pictorial support is a tool much appreciated by children and widely used in pain assessment to increase their participation and control of their care (Benjaminsson et al., 2015; Mesko et al., 2011). Two image questions were used in the PicPecc estimation template: one to select how they feel with the help of emojis and one image question to describe where on the body the nausea was experienced. There was a text question giving the children free rein to write how they felt. They were also able to design their own avatar, and the App had a reward system where they won different pets. Depending on the results of the assessment, they received various self-care tips or requests to contact the nurse. In addition, the children could observe statistics for their assessments and access a read aloud function in the App if they could not read. The App contained a note function they could activate to remind them to assess themselves.

### Data analysis

The analysis process aimed to deepen the understanding of children’s experience of using an App in nausea management. The analysis was divided into (i) nausea management and (ii) acceptability of using the App. All interviews were transcribed verbatim by the first and second author (L.E., M.O.). All text was read several times to select meaning units that answered the aim of the study. The meaning units were then organized into codes if they had similar content. All codes were unique in their content (Graneheim et al., 2017; Graneheim and Lundman, 2004). This process was conducted by two of the authors (L.E., M.O.) individually, after which they compared and discussed the codes. The authors then worked together and looked for differences and similarities between the codes, dividing them into different subcategories and thereafter categories. The last author (S.N.) read and reviewed the codes and discussed the categories with the other authors in order to fulfil trustworthiness in the analysis process (Morse, 2015).

### Ethical consideration

All the participants (including parents of children under the age of fifteen) received information about the study orally and in writing, adapted in an age-appropriate manner. Written consent was obtained from the parents of children <15 years and assent from children <15 years or consent from children ≥15 years. Confidentiality was maintained in the study by employing pseudonyms with all citations.

### Findings

A total of eleven children were asked to participate, three of whom declined. Five boys and three girls between the ages of five and fifteen (median age ten and a half years) participated in the study. The represented childhood cancer diagnoses were leukaemia, osteogenic sarcoma, Ewing sarcoma, rhabdomyosarcoma and brain tumour. After analysis and processing of the material, three categories and six subcategories Table 1 emerged.

**Table 1.** Categories and subcategories.

Categories	Subcategories
Communicating feelings	Children’s opportunity to describe feelings
	Children’s communication was not always reciprocated
Playfulness generated in motivation	The reward system stimulated children’s playfulness
	Being creative in the App arouses children’s interests
App adaptable to children’s capabilities	App adaptable for each child’s nausea management
	The questions were adaptable to the child’s choice

### Communicating feelings

This category described the App as a way for the children to communicate with healthcare staff about how they were feeling and if they needed support. The various questions in the App were also helpful to define nausea. The children were able to report both wellbeing and nausea in the App and appreciated being able to report different types of feelings.

#### Children’s opportunity to describe feelings

The use of an App gave the opportunity to reflect on feelings, both current and in the past. The children were diligent users of the statistics function in the App and found it very helpful in tracking how they had assessed themselves and how the way they were feeling had changed over time.

*‘Hmm. Yes, that’s what I thought was interesting because you can like see how you rated it before’.* Max aged 15.

The meaning of using the App varied over time according to the children’s need for nausea management, which could fluctuate greatly during treatment. Some days the nausea was better, while other days it affected them more noticeably. They also said their wellbeing could vary from hour to hour, and that nausea could sometimes arise suddenly. Moreover, certain chemotherapy drugs induced more nausea than others, and the children experienced nausea at its worst when treated with these.

*‘It depended… or I thought I can’t just rate it when I feel bad, I should rate it when I feel good’.* Linus aged 11.

The children sometimes experienced severe nausea. How they wanted to convey this to the nurses varied but they found they could convey their wellbeing more clearly and in more detail through the questions in the App. Using the App meant the children were asked fewer questions about their wellbeing but sometimes they thought it was easier to just directly tell the nurse about their nausea.

*‘I usually inform them if I feel kind of bad… I like to just say, I mean talk… but. For me it’s fine I can tell them’.* Max aged 15.

In the text question, there was more room for the children to describe their wellbeing in their own words. Although they did not always use this option, they felt it was a good help when they wanted to describe their feelings more clearly.

*‘If I like, was off food and hadn’t eaten anything… that’s when you can describe more how you feel’.* Elin aged 11.

Regarding the section in the App where the children could point out where on the body, they felt nauseous, they often selected one or more places. For example, they might choose the stomach, neck, mouth, or chest, and use a thumbs up to represent that everything was fine. The children felt the App well represented the parts of the body where they felt bad.

*‘You’ve chosen good places. It’s like where you usually feel sick. I mean I don’t feel sick in other places’.* Max aged 15.

The stomach and mouth were the most commonly selected places. However, they sometimes said they never felt bad and gave the picture a thumbs up.

*‘Thumbs up – I usually never feel sick’*. Leo aged 5.

#### Children’s communication was not always reciprocated

Although the App supported the children in highlighting their nausea, this did not always result in their receiving sufficient support from the nurses. In accessing one section of the App when assessing a high level of nausea, the children are referred to the nurse. In the interviews, they talked about how the nurses help them, especially by giving them medicine for nausea. Being helped by medicine was important, and there was more or less effective ‘nausea medicine’. However, the nurse did not always listen to the child’s wishes about what would work best.

*‘Some nurses give it to me so I can sleep and some don’t want to give it to me until later when I am supposed to sleep… oh then I sleep and then when I wake up the worst is over’.* Linus aged 11.

### Playfulness generated in motivation

The category highlighted that playfulness was necessary to motivate use of the App. The children said the App had several functions they liked, such as winning pets and receiving reminders to do their ratings. They also said they wanted to continue using the App for treatment courses in the future. However, there were several areas that could be improved and several functions that had development potential.

#### The reward system stimulated children’s playfulness

The use of a reward system was valuable and motivated the use of assessments. When the children reported how they felt, they enjoyed getting pets as a reward. This was like receiving a gift and generated excitement by the gradual reveal of the pet as they did more ratings.

*‘Oh the cat and the rabbit and the dog is almost ready… you can see its eyes’.* Leo aged 5.

The reward system was a support in helping treatment to progress. One of the older children thought the pets were good but that it might be even better for the younger children.

*‘It felt like you got further. So you felt you’d completed it’*. Elin aged 11.

#### Being creative in the app arouses children’s interests

The children appreciated the playfulness of the App and had suggestions for how to improve this. Playful elements, such as being able to choose clothes and the look of the avatar were appreciated the most, although they also thought there could be more options for their avatar, for example, more hair styles and colours.

*‘A few more hair colours’*. Linnea aged 10.

The pictures did not always fit in with the question of where on the body they feel nausea. The image of the mouth did not have the same design as the other images.

*‘The picture of the mouth was a bit strange… it should be more open and… also have red by the mouth’.* Linus aged 11.

When the children found it difficult to remember to assess themselves, the note function was helpful. For some children, the note function was not playful enough to capture their interests and on those occasions their parents were helpful in reminding them to rate themselves. On occasions when the children felt very bad, they chose to assess their nausea afterwards.

*‘I tried to remember… I got a reminder too… I mean it came up on the screen but didn’t make a noise… I could click it away’.* Linus aged 11.

### App adaptable to children’s capabilities

This category shows that children are unique and certain aspects of the App suited some children better, depending on age and level of development. They also experience different symptoms and have different ways of dealing with their nausea.

#### App adaptable for each child’s nausea management

The App allowed individualised nausea management as it was adaptable to the children’s requirements for this. When the children rated themselves, they were moved on to various self-care tips that could help them deal with their nausea, if they did not already have their own strategies for dealing with it.

*‘Hmm. have a glass of water, watch a film, have some fresh air, and then a video usually pops up and different… they helped… it helped me with… what to do when I feel sick’*. Linnea aged 10.

One piece of advice from the App was to eat simpler foods in case of nausea. This was not always well received, as the children wanted to avoid hearing the word ‘food’ when they were unwell.

*‘Not if you feel that sick – I don’t want to hear about food then’*. Linus aged 11.

The children had developed their own strategies for dealing with their nausea, so the App self-care tips were less useful to them. Everything from taking medicine, sleeping, playing, playing games, watching movies, avoiding food or eating food were methods that emerged in the children’s stories as good strategies for dealing with their nausea.

*‘I usually say to mummy and daddy, they tell me, go and lie down and rest but I don’t want to… so then I just do something… play… and then I usually feel much better’*. Liam aged 6.

#### The questions were adaptable to the child’s choice

The children said there were a number of feelings they would like to express. They explained it was not always easy to choose an emoji to represent how they felt and that sometimes they felt several emotions, for example, alert and tired at the same time. As a result, the App was adjusted to allow the children to choose several emotions at the same time.

*‘Ah that’s, that’s good. I think so – it’s good you can have more than one at the same time, like, you can be tired or feel bad or whatever so it’s not just being tired. So you know you can kind of choose several at the same time’.* Max aged 15.

The version of the App the children were given to test was set to assess nausea, but when they logged in for the first time, there were pictures of other symptoms that could be assessed. They said they wanted to rate other symptoms, such as pain as well as nausea, and wanted to be able to rate that in the App too.

*‘Nah… well, it feels like the App is half finished… There by the statistics there are more feelings I didn’t have… oh, pain. Because when I get the M medicine then later I get… when I’m home ulcers and pain in my mouth…’* Linus aged 11.

The older children said that the faces thermometer question was easy to understand, while younger children said they thought it was difficult.

## Discussion

This study evaluated children’s experience of assessing nausea via the PicPecc App and gained knowledge about how a digital platform can alleviate nausea in children undergoing chemotherapy. The findings in this study showed how an App has the potential to give the children a voice in nausea management. The findings also point out the keys to success, for example, playfulness and adaptability, and that these key aspects have the potential to be generalized to other Apps. Other research has shown that listening to children’s wishes is important for strengthening their autonomy and that they find it valuable to be asked how they want to be involved in their care (Jibb et al., 2018; Lea et al., 2019). Coyne and Kirwan (2011) have shown that children in hospitals appreciated becoming involved in their care. The right information, participation and a creative/playful environment increased the child’s positive experience of hospital care.

Through the PicPecc App, the child was given a new way of participating in their care – having responsibility for assessing their nausea and control over how they wanted to convey this. By using the App, the child had the power to decide when and how the assessment should be made. Nevertheless, being able to describe their nausea and expressed need for help through the App did not always result in a response from the nurses. The nurses did not always pay attention to the child’s wishes, and instead supported the child the way they saw fit themselves. This clearly shows – from the child’s perspective – how children want to be treated in a person-centred way (Thunberg et al., 2021).

The PicPecc App was a helpful tool, as it was adapted according to age and acted as a support during the different phases of treatment the children underwent. They also appreciated the flexibility of the App in shifting between self-care and professional support and that it gave them opportunity to be active in decision-making. In another study by Carlsson et al. (2018) health care staff were interviewed about what they thought participation included in paediatric care. Children were usually involved but, in most cases, this was limited to smaller decisions and less time-consuming steps. According to the nurses in the study, working according to the child’s wishes and working from a medical perspective was a difficult balancing act. Many times, it was more time-efficient to work from a medical perspective, although this unfortunately affected how the children experienced their care and reduced their feeling of involvement (Carlsson et al., 2018).

The PicPecc App is a first attempt at using digitalisation with the aim of increasing person-centred care. Even if more development is needed, this is the first study to highlight the challenges and opportunities, for example, the children wanting to develop playfulness in the App to motivate self-assessments. Bernier Carney et al. (2021) also highlight the value of using gamified Apps, which contain elements of creative approaches for the child.

In person-centred care, the patient’s story and partnership between the patient and the nurse are essential (Thunberg et al., 2021). Some children found it easy to talk to the nurse, while others found it difficult. Listening to the children’s wishes to be cared for is part of person-centred care (Lea et al., 2019; Thunberg et al., 2021). The partnership between the nurse and the child is based on the formation of trust and a relationship taking shape (Thunberg et al., 2021). According to ( Gondek et al., 2017). the path to such a partnership and person-centred care in paediatrics is that the nurse must listen, respect, value and learn to understand the child’s situation and needs. When the nurse has won their trust and developed this relationship, the children have an easier time communicating their story and needs. Carlsson et al. (2020) highlights that an eHealth tool may contribute to getting children involved in the communication.

This study showed that using the PicPecc App increases opportunities for children to communicate their story to healthcare staff. According to Coyne et al. (2016), strengthening the child’s perspective involves seeing the child as competent in expressing their experiences themselves. The partnership is about the child and the nurse sharing their experiences and knowledge, with the child as an expert on themselves and their needs, sharing this with the nurse through their story (Thunberg et al., 2021). Based on this, good care can be discussed and planned (Lea et al., 2019). O’Sullivan et al. (2018) addresses how children who were too shy to say how they felt used the App to convey their symptoms. These findings are similar to that of our study, where the children said how nice it was to be able to indicate nausea in the App, as it meant healthcare staff and parents did not have to frequently ask about nausea. With the help of the App, they were able to take control of the situation and convey their feelings in the way they wanted. In person-centred care from the child’s perspective, it is important the specialist nurse respects and listens to the child regarding how they want to communicate their feelings.

Although the children in this study thought the App was a good tool to use during treatment, they had different experiences of using it. Depending on age and whether strategies for dealing with nausea had already been developed, certain aspects of the App were better or worse suited to different individuals. Children are unique and experience treatment according to their conditions. During the interviews it emerged that they had their own strategies for dealing with their nausea and wellbeing. Linder et al. (2018) have also highlighted how children with cancer developed their own strategies for managing their symptoms. Rodgers et al. (2012) have shown that distraction is a well-known way of dealing with nausea, and that children also want guidance from healthcare staff regarding strategies for managing nausea and other symptoms. Our study confirms the usefulness of distraction and that children appreciate receiving help and guidance in managing their symptoms.

### Limitations

This study has some limitations. A major limitation is the small sample size, which limits the ability to transfer the result to other contexts. The study should be repeated in a large study population. However, the purpose of a qualitative study is not to create generalizable knowledge but rather a diversity of experiences. There is a risk of population selection bias, as the children were recruited by the healthcare staff who were treating them. The fact that children who did not master the Swedish language were excluded is seen as a weakness in the study. As connecting to the video link required technical knowledge, parents were in the background during the majority of the interviews. The parents were instructed to take a passive role, although in some interviews the parents were more active. The parents’ comments and thoughts were valuable but were not analysed into the results, as the aim was to highlight the children’s experiences.

### Implications for practice

The children thought it was valuable to assess how they felt during the chemotherapy treatment, highlighting the need for these assessments to be implemented in clinical practice with routines for following up assessments with treatment alternatives. The playfulness in the App also influenced them, and they appreciated the reward system with different pets. Using the PicPecc App has the potential to increase healthcare staff’s understanding of how the child experiences nausea when he/she undergoes chemotherapy.

## Conclusion

The study showed how the PicPecc App has helped children to manage their nausea and communicate this to the nurses in different ways. Moreover, it has opened up new opportunities for participation and symptom assessment of nausea. It was evident from the children’s stories that they experienced their treatments in unique ways. The children in this study had come a long way in their treatments. If children have access to the App early in their treatment, they can receive guidance from the beginning about how to manage their nausea, reducing the need to find their own strategies.

## Supplemental Material

Supplemental Material - ‘Do you feel well or unwell?’ A study on children’s experience of estimating their nausea using the digital tool PicPecc Click here for additional data file. Supplemental Material for ‘Do you feel well or unwell?’ A study on children’s experience of estimating their nausea using the digital tool PicPecc by Linda Esplana, Malin Olsson and Stefan Nilsson in Journal of Child Health Care
