# Supplementary material for: ‘Do you feel well or unwell?’ A study on children’s experience of estimating their nausea using the digital tool PicPecc
Source: J Child Health Care. 2022 Apr 22;27(4):654–66. doi: 10.1177/13674935221089746 (PMC10676616; doi:10.1177/13674935221089746)
Supplement: Supplemental Material - ‘Do you feel well or unwell?’ A study on children’s experience of estimating their nausea using the digital tool PicPecc [file sj-pdf-1-chc-10.1177_13674935221089746.pdf]

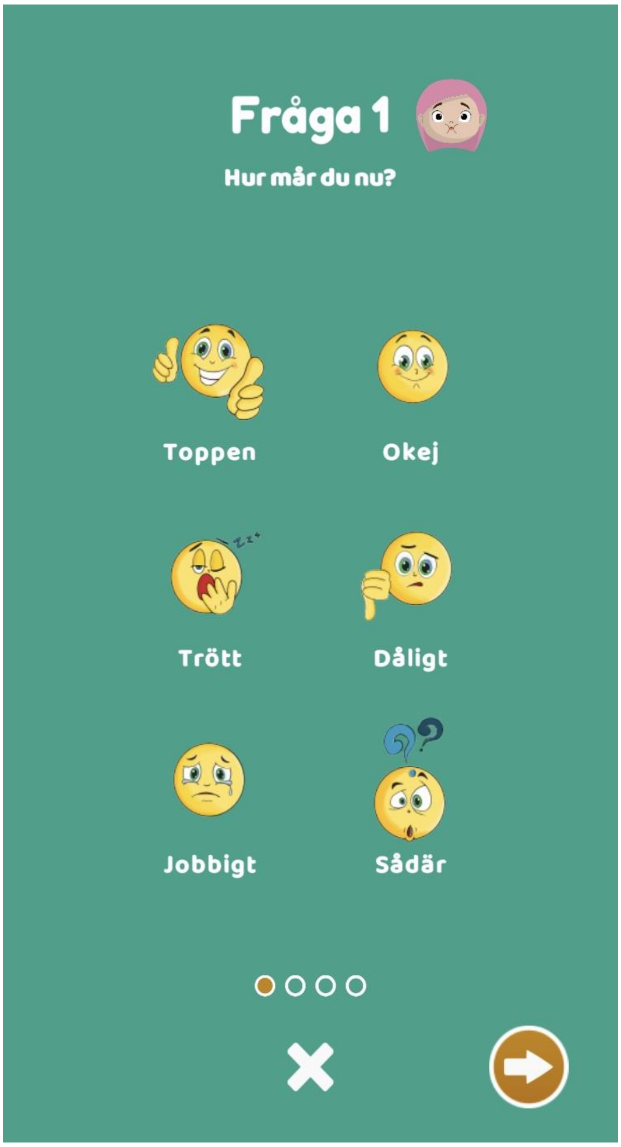
Fråga 1
Hur mår du nu?
Toppen
Okej
Trött
Dåligt
Jobbigt
Sådär

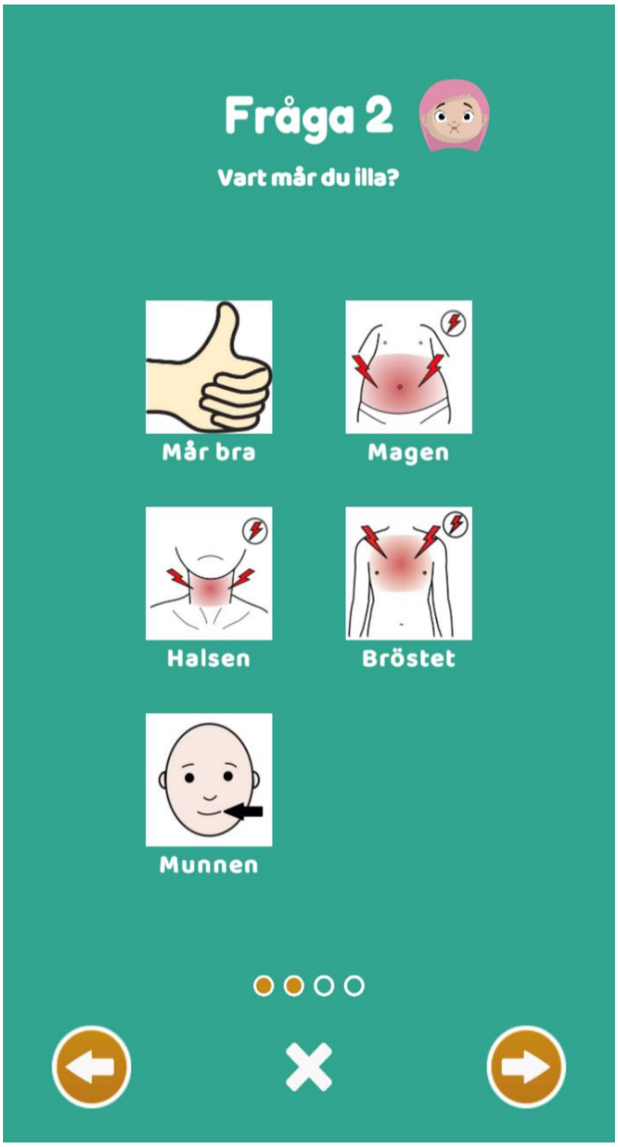
Fråga 2
Vart mår du illa?
Mår bra
Magen
Halsen
Bröstet
Munnen

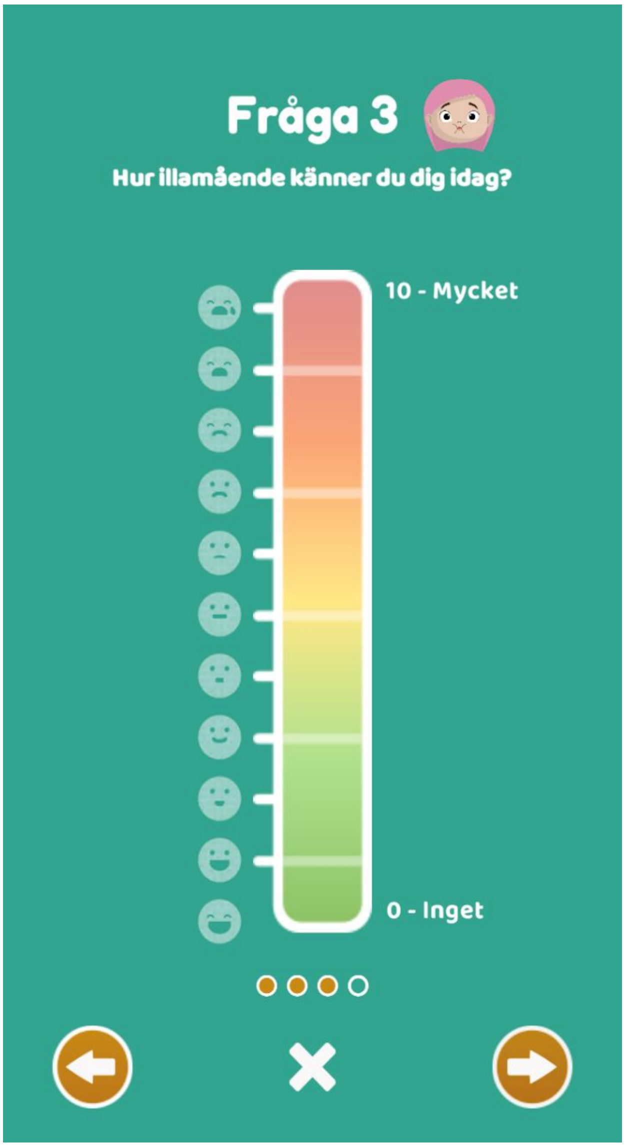
Fråga 3
Hur illamående känner du dig idag?
10 - Mycket
0 - Inget

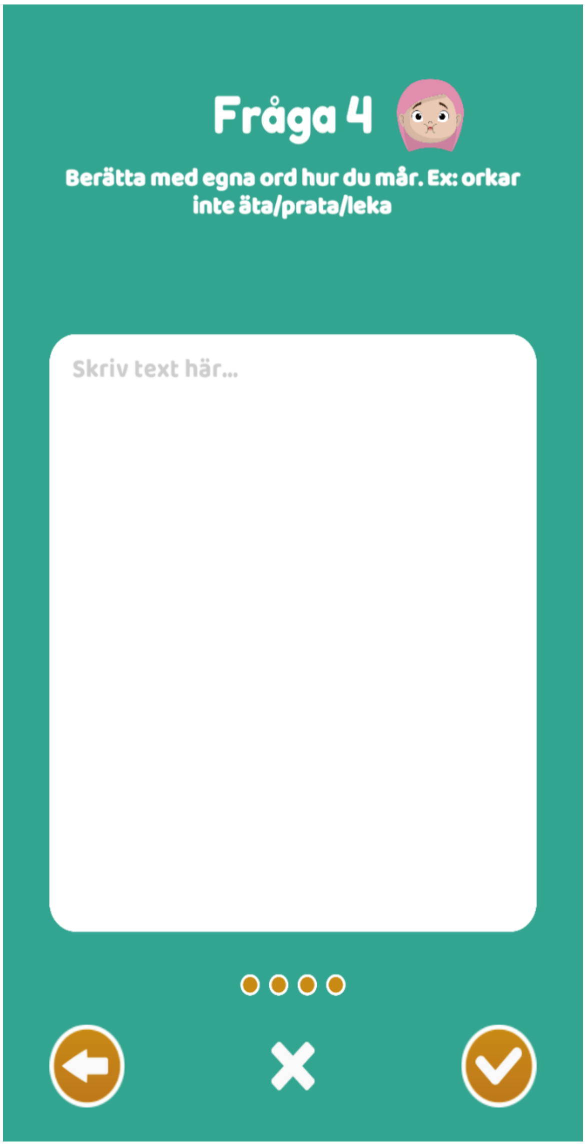
Fråga 4
Berätta med egna ord hur du mår. Ex: orkar inte äta/prata/leka
Skriv text här...

Kämpa på!

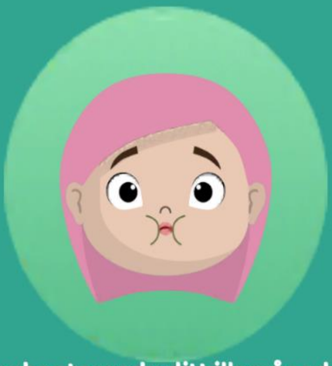

Hur hanterar du ditt illamående?
Ibland kan det hjälpa med:

Tänka på något annat
Se en film
Ät en glass
Drick vatten
Ta frisk luft

Gå till extern länk

Har du något annat kul att titta på?
